# Supplementary material for: Prevalence and risk factors of short birth interval in Bangladesh: Evidence from the linked data of population and health facility survey
Source: PLOS Glob Public Health. 2022 Apr 22;2(4):e0000288. doi: 10.1371/journal.pgph.0000288 (PMC10021594; doi:10.1371/journal.pgph.0000288)
Supplement: Attachment — Submitted filename: Response to Editors and Reviwers.pdf [file pgph.0000288.s001.pdf]

Reviewers' comments and author's responses

**Reviewers' comments**	**Authors' response**	**Reference**
**Reviewer #1**		
Thank you to the authors for the opportunity to read and review the article. Some comments for consideration: The major contribution from this paper is really that living closer to health facilities is a strong protective predictor of SBI. To strengthen this aspect of the paper, I would restructure the paper to make this the focus of the analysis. This would include writing more about this point in the introduction, discussing what other studies have found on the matter and perhaps doing more decomposed analysis focusing on this.	We have edited the introduction section and critically revised the predictors of SBIs that have been identified so far in LMICs. As suggested, we now offer more focus to the health facility level factors. The edited relevant sections now read- *The occurrence of SBI is mostly associated with the women's lack of understating about its adverse effects on their own health as well as their children's health and lower access to and use of family planning and contraception services [14–18]. Multidimensional factors affect access to healthcare services, including individual-, household-, community-, and health facility-level factors need to be studied to identify the key determinants of SBI [20]. Many of these factors are intertwined and need to be examined together. However, there is scarce literature particularly in the context of LMICs.*	5

Previous studies on SBI considered only a limited set of confounding factors, mainly the individual-level factors such as women's age, age at marriage, number of children given birth, nutritional status, educational achievements and economic conditions of the household, etc. [17,21–23]. These factors are also intricately connected to inadequate access to health care services, particularly family planning and contraception services. Community-level norms such as having children soon after marriage, having several children to support family income in the future and/or safety reasons, desire for a child of a particular sex (particularly the male child)[15,25–27] also play important determinants of SBI.

Essentially, access to family planning and contraception services are influenced, to some extent, by health facility-level factors, including the availability and quality of family planning and contraception services near the women's residence [27]. This access can play an important role

	in reducing SBI by reducing the individual-level barriers particularly among disadvantaged women [28]. However, there exists little relevant research in this regard, mainly due to the lack of population- and health facility-level data. Although a few studies considered the distance of women's residence from the nearest healthcare facilities, they provide an incomplete understanding of the associations between health facility-level factors and SBI [21,22]. Methodological challenges of the existing studies are also common, including small datasets [19] and ignoring the hierarchical structure of data where single-level analysis produces imprecise results [10,11,13,20]. Using nationally representative population- and health facility-level data and advanced statistical modelling, this study examines the individual-, household-, community-, and health facility-level factors associated with SBI in Bangladesh.	
It is also key that this be structured within the existing theory of health care access. Perhaps the authors could refer to	We thank the reviewer for introducing us with the Levesque et al.'s paper which was really an interesting reading. We have restructured the introduction section following this model. Please see our response above.	

Levesque et al.'s theory on access to healthcare: https://link.springer.com/article/10.1186/1475-9276-12-18/ This discussion could include a stipulation of the potential transmission mechanisms as well.		
The authors state that the analysis is cross-sectional, but with the number of observations, I would recommend they use a matching technique to say something causal about the relationship.	Since we merged PSUs instead of individuals with the nearest health facilities, the use of the matching technique reduce the study sample size significantly. We, therefore, did not go for matching. However, we agree that our present analysis produces correlational findings rather than casual which we clearly mentioned in the study limitation section. This reads-<br>*However, a limitation of this study is its cross-sectional data; therefore, the associations are correlational rather than causal.*	16
Page 4: "Like many other LMICs, this high number of births in short intervals	We have corrected this sentence.	4

caused a large-scale of several adverse health outcomes". There seems to be a word missing after large-scale.		
Page 4 "which will require substantially reduce the prevalence of births in SBI through initiating policies and programmes". Should this be 'require a substantial reduction'?	We have corrected this sentence.	5
The research question needs to be more clearly stated in the end of the Introduction section.	We have edited the relevant sentence to clearly state the research question. This now reads-<br>*Using nationally representative population- and health facility-level data and advanced statistical modelling, this study examines the individual-, household-, community-, and health facility-level factors associated with SBI in Bangladesh.*	6
The authors seem to be using different fonts on page 6.	Thanks for bringing this to our attention. We have fixed this now.	

It is unclear to me how the two datasets will be linked. Are you able to link individuals from the two data sets to one another? I realised later that you are linking individuals to data on their closest health facility, but this should be made clear in the data section.	We have added a paragraph in the methods section to make this clear. It reads-<br>*The GPS point locations are available for each of the 672 PSUs included in the 2017 BDHS and 1,524 healthcare facilities included in the 2017 BHFS. We linked them, i.e PSUs with the nearest healthcare facilities, using the administrative boundary linkage method. The detail for this method has been published elsewhere [23,24].*	7
What is a Upazila?	Upazila is an administrative region in Bangladesh, functioning as a sub-unit of a district. We have now made this clear in the manuscript.	11
Page 6: "The inclusion criteria were i) had at least two pregnancies" - there seems to be a word missing here.	We have corrected this. It now reads-<br>*The inclusion criteria were i) women had at least two pregnancies, of which the most recent one occurred within five years of the survey date and ended with a live birth……….*	8
Page 7 "survival status of the child born from the second most pregnancy" -	Thanks for bringing this to our attention, we have corrected it now.	9

should this be 'most recent'?		
Page 12 "but it is in the range of global range" - perhaps do not use range twice.	We have corrected this.	13
Page 13 "they have a lower probability of getting pregnancies inshort intervals." - should this be "having pregnancies"?	We have corrected this.	14
**Reviewer#2**		
**Summary**		
The authors assessed the predictors of short birth interval in Bangladesh by linking two national databases. Using descriptive and multilevel analysis, the study found that individual, household, and community level factors, and availability of modern contraceptives at health facility were associated with short birth interval.	We have addressed these comments in this revised version of the manuscript. Please see our response to the first comment of the first reviewer and your comment on the methods section. Language has also been edited in this revised version to improve readability.	

This study is relevant given that short birth interval remains a leading cause of maternal and child mortality in developing countries. A strength of this study is the nationally representative data. However, the authors did not provide literature to support healthcare facility-level factors and short birth interval in the Introduction. Moreover, the selection of potential predictors was not based on adequate evidence from systematic reviews. I suggest the authors rewrite the Introduction to include the relevance of healthcare facility level factors and short birth interval. The language is unclear. I advise that the authors revise the language		

to improve the readability.		
**Major revision**		
**Introduction**		
1. The authors should rewrite this section to integrate the relevant literature on predictors of short birth interval, healthcare facility-level factors, and short birth interval.	The first reviewer had a similar comment. We have revised the introduction section where we critically revised the predictors of SBI and focused on the health facility-level factors. Please see our response to the first reviewer's first comment.	
**Methods**		
2. The list of potential determinants was not comprehensive. Other factors e.g., breastfeeding, the sex of previous child have been found to be the only significant factors in a recent systematic review published by Pimentel et al. BMC Pregnancy and Childbirth 2020. The paper	The potential determinants for this study were selected comprehensively in three stages. We have now clarified this issue in the exposure variable section by adding a few sentences. The edited section now read-<br><br>*A comprehensive literature search was conducted in five databases (Medline, Embase, Web of Science, CINHAL, and Google Scholar) to identify potential determinants of SBIs [10,11,13–18,20]. The factors*	8

could be strengthened by including these factors in the models.	*identified and later found available in the dataset were considered. Later their associations with the SBIs were tested using the forward regression approach. Factors found significant at the p=0.20 level were retained in the analysis. Finally, these factors were then summarised as individual-, household-, community-level factors following the socio-ecological model of health [36].*<br><br>However, we acknowledge that some factors that were found significant in other studies are still not considered. This is because either these specific variables were found insignificant for our data in the forward regression model, or they were not available in the dataset we analysed.	
**Discussion**		
3. How does the findings on healthcare facility relate to the literature?	We have now added the following paragraph in the discussion section to explain the effects of health facility-level factors on SBIs.<br>*Provision of modern contraceptives at the nearest health facility from women's houses is a strong negative predictor of SBI. Also, the*	16

	prevalence of SBI increases with an increasing average distance of the nearest health facilities from women's residences. These relationships are reasonable, as the provision increases the availability and the smaller distance increases the accessibility [71]. Service convenience is crucial particularly in the post-partum period when movement is considered risky. Women in Bangladesh usually face restrictions in travelling long distances and these restrictions are linked with gender, community norms, transport and time barriers.	
**Minor revision**		
**Abstract**		
4. The authors stated "The Sustainable Development Goals target significant reductions….." The authors should be specific by stating which goal(s).	We have edited the relevant sentence as follows:<br>*The Sustainable Development Goals 3 targets significant reductions in maternal and under-five deaths by 2030.*	2
5. They should present additional information on the sample (respondents),	We have revised the methods section of the abstract to address this comment. The revised section now reads-	2

the inclusion criteria, and sample size.	*A total of 5,941 women included in the 2017/18 Bangladesh Demographic and Health Survey 2017/18 and 1,524 healthcare facilities included in the 2017 Bangladesh Health Facility were linked and analysed. The sample was selected based on the availability of the birth interval data between the two most recent subsequent live birth. SBI was defined as an interval between consecutive births of 33 months or less, as recommended by the World Health Organization and was the outcome variable. Several individual-, households-, and community-level factors were considered as exposure variables. We used descriptive statistics to summarise respondents' characteristics and multilevel Poisson regression to assess the association between the outcome variable with exposure variables.*	
6. Provide statistics with 95% CI on education and rural women	Women's education and the location of their residence (urban or rural) had statistically insignificant associations with SBI.	2
7. "The prevalences of SBI were higher among women for whom the children born	We have revised this sentence. This now reads-<br>*The prevalences of SBI were higher among women whose second most*	2

from the second most pregnancies died" This statement is unclear. Please revise.	*recent child died (PR, 5.23; 95% CI, 4.18-6.55),……*	
8. In the conclusion, the authors should be specific and indicate who the targets of interventions should be, based on the findings from the study.	We have now revised the relevant sentence. This now reads- *Targeted and tailored regional policies and programmes are needed to increase the awareness of SBI and associated adverse health outcomes and availability of modern contraception in the healthcare facilities.*	3
**Introduction**		
9. State the specific goals and objective that target maternal and child mortality.	We have added this.	6
10. The authors provided the prevalence of SBI as 26% in the introduction. How different is this statistic from what has been presented in the results?	We have revised the relevant sentence in the introduction section to make it different from this study finding. The revised sentence now reads- *In Bangladesh, around a quarter of pregnancies that end with live births are born in short intervals and the total number equates to over a million live births in a year [8]*	4
**Methods**		

11. Ethics statement is missing in the text. I advise they include statement on ethics consideration as well as describe the process of obtaining permission to access the data.	As per the journal's guideline, this statement has been included at the end of the manuscript.	17
12. Statistical analysis: Could the authors describe how they handled missing data in the analysis?	Of the 5,941 participants from the original sample of 20,127 were included in our analysis. The missing data, which were less than 0.5%, were automatically excluded in our regression analysis.	
13. How were the exposure variables selected?	We have added the following sentences in the exposure variable section of the methods to make this clearer. Relevant sentences now read-<br>*A comprehensive literature search was conducted in five databases (Medline, Embase, Web of Science, CINHAL, and Google Scholar) to identify potential determinants of SBI [10,11,13–18,20]. The factors identified in the literature search and later found available in our dataset were considered. Later their associations with the SBI were tested using the forward regression approach. Factors found significant*	8

	at the p=0.20 level were retained in the analysis. Finally, these factors were then summarised as individual-, household- and community-level factors. Individual-level factors were……….	
14. What level of significance was considered for an exposure variable to be retained in the analysis?	We have now made this clearer in the revised sentence. Please see our response above.	
**Results**		
15. "Distribution of short birth intervals across socio-demographic characteristics" indicate where to find this in the manuscript	We have now cited the relevant table number in the text description.	11
16. The prevalence of short birth interval had been reported in the Introduction. It is not clear why the authors included existing statistics in the results. They should provide information to justify the decision.	We have revised the relevant sentence in the introduction section to make it different from this study finding. The revised sentence now reads-<br>*In Bangladesh, around a quarter of pregnancies that end with live births are born in short intervals and the total number equates to over a million*	4

	live births in a year [8].	
**Discussion**		
17. This contradicts the information in the abstract where richest wealth quintile had lower odds of SBI. This should be revised.	Thanks for bringing this in our attention. We have fixed this now.	15
